# Pregnancy Rhinitis: Pathophysiological Mechanisms, Diagnostic Challenges, and Management Strategies—A Narrative Review

**DOI:** 10.3390/life15081166

**Published:** 2025-07-23

**Authors:** Cristina Stefania Dumitru, Flavia Zara, Dorin Novacescu, Diana Szekely, Dan Iovanescu, Gheorghe Iovanescu, Raul Patrascu, Catalin Dumitru

**Affiliations:** 1 Department II, Discipline of Histology, ‘Victor Babes’ University of Medicine and Pharmacy Timisoara, Eftimie Murgu Square, No. 2, 300041 Timisoara, Romania; cristina-stefania.dumitru@umft.ro (C.S.D.); flavia.zara@umft.ro (F.Z.); novacescu.dorin@umft.ro (D.N.); 2 Doctoral School, ‘Victor Babes’ University of Medicine and Pharmacy Timisoara, Eftimie Murgu Square, No. 2, 300041 Timisoara, Romania; diana.szekely@umft.ro; 3 Otorhinolaryngology Department, ‘Victor Babes’ University of Medicine and Pharmacy Timisoara, Eftimie Murgu Square No. 2, 300041 Timisoara, Romania; dan.iovanescu@umft.ro (D.I.); giovanescu@umft.ro (G.I.); 4 Department of Functional Sciences, ‘Victor Babes’ University of Medicine and Pharmacy Timisoara, 300041 Timisoara, Romania; 5 Department of Obstetrics and Gynecology, ‘Victor Babes’ University of Medicine and Pharmacy Timisoara, Eftimie Murgu Square 2, 300041 Timisoara, Romania; dumitru.catalin@umft.ro

**Keywords:** pregnancy rhinitis, nasal obstruction, estrogen, vascular congestion, maternal health

## Abstract

Pregnancy rhinitis (PR) is a transient, non-infectious nasal condition affecting a significant number of pregnant women, yet often remains underdiagnosed or misclassified. It can substantially impact maternal quality of life, sleep, and even fetal oxygenation. This narrative review explores the current understanding of PR, including hormonal and vascular mechanisms, clinical criteria, and therapeutic approaches considered safe during pregnancy. Despite increasing recognition, the differentiation between PR and other rhinitis forms remains challenging. Limited therapeutic options and the absence of standard diagnostic guidelines further complicate management. Evidence supports a multifactorial etiology involving estrogen, progesterone, and placental growth factors. Non-pharmacologic strategies are first-line, while pharmacological interventions are cautiously employed. PR is a distinct and clinically relevant condition requiring increased awareness among ENT and obstetric professionals. Future research should focus on standardized diagnostic criteria and evidence-based treatment protocols to improve maternal–fetal outcomes.

## 1. Introduction

Pregnancy rhinitis (PR) represents a distinct clinical entity characterized by nasal congestion arising during pregnancy, in the absence of allergic or infectious triggers, and resolving shortly after delivery. Despite its transient nature, PR can significantly impair maternal quality of life, disturb sleep patterns, and may be associated with adverse perinatal outcomes through mechanisms related to chronic hypoxia and fragmented rest. The condition is often overlooked in both otorhinolaryngologic and obstetric evaluations, leading to underreporting and insufficient clinical management [1].

The pathophysiological mechanisms underlying PR are not fully elucidated but are believed to involve complex hormonal influences—particularly elevated levels of estrogen and progesterone—as well as enhanced vascular permeability and mucosal edema [2]. These alterations affect nasal airflow and mucociliary function, contributing to the persistent congestion experienced by affected individuals [3,4]. Although several observational studies and clinical reports have explored the epidemiology and clinical characteristics of PR, diagnostic criteria remain poorly defined and are not universally adopted in clinical practice [5].

From a therapeutic standpoint, the management of PR poses a challenge due to the limited safety data for pharmacologic interventions during pregnancy. Non-pharmacological strategies are often recommended, yet their efficacy is variable and not well-documented in the literature. Moreover, the differentiation between PR and other forms of rhinitis—including allergic, non-allergic vasomotor, or medication-induced types—is essential for appropriate clinical decisions but remains difficult in the absence of validated diagnostic tools [6].

Although this is a narrative review, the relevant literature was identified through searches in PubMed, Scopus, and Web of Science databases, using keywords such as ‘pregnancy rhinitis’, ‘nasal obstruction in pregnancy’, and ‘hormonal rhinitis’. The search included articles published between 2000 and 2025 and focused on both clinical and mechanistic aspects of the condition.

Given these considerations, this narrative review aims to synthesize current knowledge on the pathogenesis, diagnostic approach, and management options for PR, highlighting gaps in the literature and proposing directions for future clinical and research initiatives.

## 2. Epidemiology and Risk Factors

Pregnancy rhinitis (PR) is a common but under-recognized condition characterized by persistent nasal congestion during pregnancy, typically emerging in the second or third trimester, with symptom resolution by two weeks postpartum [1]. Estimates of prevalence vary widely due to inconsistent definitions and methodologies, generally ranging between 9% and 39%. For instance, a Polish study found that 39% of pregnant women between 13 and 21 weeks of gestation experienced symptoms consistent with pregnancy rhinitis [7].

The variability in reported prevalence largely reflects the absence of universally accepted diagnostic criteria. Some studies rely on self-reported nasal congestion, while others apply stricter definitions that exclude women with a history of allergic rhinitis, upper respiratory infections, or structural nasal abnormalities [1,8]. Furthermore, many investigations fail to distinguish pregnancy-induced rhinitis from pre-existing conditions exacerbated during gestation. This diagnostic ambiguity has contributed to both underreporting and a limited understanding of the true burden of the disease [9].

Despite these methodological limitations, several trends have emerged consistently across the literature. First, pregnancy rhinitis appears to be more prevalent in the later stages of pregnancy [7]. Most studies report a progressive onset of symptoms, with the majority of cases developing after the 20th week of gestation [6,10]. This timing corresponds with the gradual elevation in circulating levels of estrogen and progesterone, which are thought to play a key role in the pathophysiology of nasal congestion during pregnancy [1].

A noteworthy aspect of pregnancy rhinitis is its occurrence in women without any prior history of chronic nasal symptoms or allergic disease. Many affected patients report having never experienced nasal obstruction or rhinorrhea outside of pregnancy. This distinguishes PR from allergic or non-allergic rhinitis and may contribute to diagnostic confusion, especially in primary care or obstetric settings where nasal complaints are often attributed to benign or transient causes. As a result, the condition is frequently overlooked, and symptomatic relief may be delayed or neglected entirely [5,11].

In addition to gestational age, several demographic and clinical factors have been investigated as potential risk indicators for pregnancy rhinitis (PR). One of the most frequently cited is increased body mass index (BMI). A Turkish observational cohort study including 167 pregnant women reported a significant positive association between higher BMI and severity of nasal congestion [12]. Similarly, a cohort from Iraq including 146 pregnant women found that high BMI was a statistically significant risk factor for PR (*p* = 0.001), particularly in the first trimester [13].

Tobacco exposure—either active smoking or secondhand smoke—has also been identified as a contributing factor. Tobacco use is known to impair mucociliary clearance and exacerbate nasal inflammation, which may potentiate the effects of hormonal vasodilation and mucosal edema during pregnancy. While smoking is generally discouraged in pregnancy due to broader fetal risks, its role in nasal pathology provides an additional rationale for cessation counseling in symptomatic patients [14,15].

Other investigated factors include multiparity, maternal age, and family history of rhinitis. However, findings remain inconclusive: a comprehensive review found no association between maternal age, parity, or fetal sex and pregnancy-induced rhinitis [1]. It is also unclear whether pregnancy-related comorbidities influence the development or progression of PR, as these variables have not been consistently examined in large-scale cohorts. Interestingly, the season of symptom onset has also been explored, particularly in studies conducted in temperate climates. Some authors have proposed that seasonal variations in humidity and temperature may amplify baseline mucosal reactivity, although this remains speculative and difficult to isolate from environmental allergens and infections [16].

In summary, although the precise prevalence of pregnancy rhinitis remains uncertain due to diagnostic inconsistencies, the condition is clearly more common than currently recognized. It tends to emerge in the second or third trimester, in otherwise healthy women without a history of chronic rhinitis. Risk factors such as elevated BMI and smoking may exacerbate symptoms, and increased awareness among obstetric and ENT professionals is essential to improve recognition and management. Future epidemiologic studies should focus on applying standardized diagnostic definitions and accounting for potential confounders to better estimate the burden of disease and its modifiable determinants.

## 3. Pathophysiology

Pregnancy rhinitis is considered a multifactorial condition resulting from the complex interplay between hormonal, vascular, and mucosal changes during gestation. Unlike allergic or infectious rhinitis, this form of nasal congestion develops in the absence of identifiable triggers, suggesting that systemic pregnancy-related changes play a central role in its pathogenesis. Elevated levels of estrogen, progesterone, and placental hormones are thought to contribute significantly by modifying the structure and function of the nasal mucosa. In addition to endocrine influence, alterations in blood volume and vascular permeability, as well as functional changes in ciliary activity and mucosal glandular secretion, have all been implicated [17]. Understanding these mechanisms is essential for differentiating pregnancy rhinitis from other nasal disorders and for guiding both diagnosis and management.

### 3.1. Hormonal Mechanisms

The influence of hormonal fluctuations during pregnancy represents the keystone of current hypotheses regarding the development of pregnancy rhinitis. Among the most implicated hormones is estrogen, which plays a key role in vascular remodeling and mucosal hyperreactivity. Estrogen receptors have been identified within the nasal mucosa, and rising serum levels during the second and third trimesters have been shown to correlate with symptoms of nasal obstruction [18]. Estrogen enhances vascular permeability, induces edema of the submucosa, and upregulates histamine receptors, contributing to the sensation of nasal blockage even in the absence of inflammation.

Progesterone, although traditionally recognized for its smooth muscle–relaxing effects in the uterus, also has significant vasodilatory action in the upper respiratory tract. By reducing vascular tone and increasing plasma volume, progesterone contributes indirectly to mucosal swelling in the nasal passages. Furthermore, placental growth hormone (PGH) and other gestational peptides such as vascular endothelial growth factor (VEGF) may further promote mucosal congestion by stimulating angiogenesis and increasing vascular surface area within the nasal turbinates [19].

In addition to their vascular actions, pregnancy-associated hormones—particularly estrogens—may influence nasal glandular activity through neuromodulatory pathways. Estrogen receptors (ERα and ERβ), abundantly expressed in the hypothalamus and involved in the regulation of vasopressin release, play a central role in fluid and electrolyte homeostasis [20]. It is plausible that similar estrogen-mediated mechanisms could affect mucosal hydration and secretory behavior at the level of the nasal epithelium. Increased estrogen concentrations during pregnancy may enhance secretomotor activity in nasal glands, leading to hypersecretion of mucus and contributing to congestion. These symptoms are typically transient and resolve shortly after delivery, supporting the hypothesis that hormonal modulation of neuroendocrine circuits—possibly via estrogen-sensitive vasopressin pathways—underlies the pathophysiology of pregnancy rhinitis.

Importantly, not all pregnant women experience rhinitis, suggesting the existence of individual susceptibility, possibly mediated by genetic factors, pre-existing nasal reactivity, or variations in receptor sensitivity. Further studies are needed to clarify these predispositions and to quantify the threshold levels at which hormonal effects translate into clinical symptoms.

### 3.2. Vascular and Structural Changes

The nasal mucosa is highly vascularized and sensitive to changes in blood flow, which renders it particularly susceptible to the hemodynamic and structural adaptations occurring during pregnancy. As gestation progresses, the maternal cardiovascular system undergoes significant adjustments, including increased cardiac output, expanded plasma volume, and reduced systemic vascular resistance. These changes, though physiologically beneficial for uteroplacental perfusion, can inadvertently contribute to venous engorgement and mucosal edema in the upper airway, particularly within the nasal turbinates [21,22].

One of the hallmark features observed in pregnancy rhinitis is nasal turbinate hypertrophy caused by vascular congestion and increased permeability of the subepithelial capillaries. Histological examinations have demonstrated mucosal thickening and dilated venous sinusoids within the lamina propria, suggestive of a passive vascular mechanism rather than an active inflammatory process [23]. These changes may be further potentiated by hormonal vasodilators such as relaxin, which acts synergistically with estrogen to reduce vascular tone.

Additionally, the capillary leak phenomenon, a well-described consequence of estrogen-mediated endothelial modulation, may lead to transudation of plasma into the interstitial space, worsening mucosal swelling. The resultant airflow resistance is particularly notable during the night, when patients are recumbent, explaining the frequent association between pregnancy rhinitis and nocturnal breathing difficulties or snoring [24].

Structurally, the mucosa may also exhibit increased glandular density and hyperplasia, leading to an augmented secretory response, even in the absence of infection or allergy. These changes may manifest clinically as rhinorrhea, contributing to patient discomfort and reduced quality of life [25]. However, the absence of typical inflammatory markers, such as eosinophils or neutrophils, differentiates pregnancy rhinitis from allergic or infectious forms.

**Figure 1:**
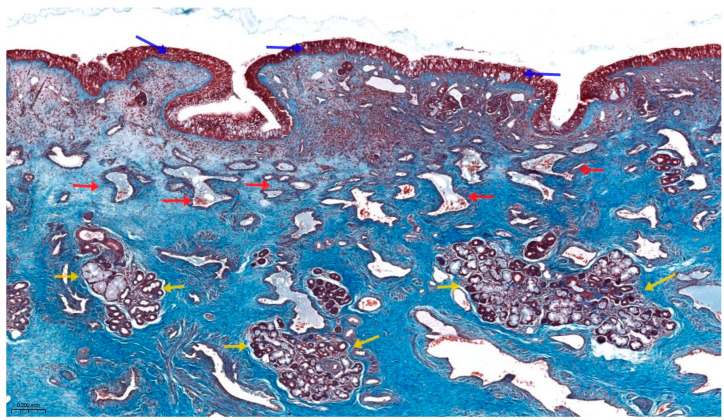
Transversal section of a human nasal turbinate stained with Masson’s trichrome. The respiratory epithelium (blue arrows) overlays a thickened lamina propria rich in congested blood vessels (red arrows) and enlarged mucoserous glands (yellow arrows). Image acquired at 10× magnification.

These histological alterations are illustrated in Figure 1, which presents a transversal section through a human nasal turbinate stained using Masson’s trichrome technique. The image highlights the thickened respiratory mucosa, subepithelial vascular congestion, and glandular hyperplasia within the lamina propria—features consistent with the histopathological substrate of pregnancy rhinitis. This specimen originates from the internal archive of the Histology Department, “Victor Babeș” University of Medicine and Pharmacy Timișoara, and illustrates the connective tissue remodeling and vascular dilation responsible for nasal obstruction in affected individuals.

Another important aspect is the potential impact on mucociliary clearance. Edema of the epithelial lining and disruption of ciliary beat frequency—possibly due to altered pH or osmolarity in the nasal secretions—can lead to impaired mucosal function. This may increase the risk of secondary infection or sinus congestion, although overt sinusitis remains rare in uncomplicated cases of PR [26].

The anatomical configuration of the nasal cavity also plays a role. Narrow nasal passages or pre-existing mild turbinate hypertrophy may become clinically significant under the influence of pregnancy-induced changes. Some authors have proposed that these structural susceptibilities, when combined with the physiological alterations of pregnancy, may explain why only a subset of women develop rhinitis symptoms [27].

In summary, vascular and structural changes during pregnancy significantly contribute to the development of nasal congestion, primarily through mucosal engorgement, increased permeability, and altered airway architecture. These mechanisms, driven largely by systemic and local hemodynamic shifts, reinforce the importance of recognizing pregnancy rhinitis as a distinct clinical condition rather than a variant of existing nasal disorders.

### 3.3. Functional and Neurophysiological Modifications

Beyond hormonal and vascular mechanisms, functional alterations in nasal physiology and local neurogenic regulation also contribute significantly to the clinical manifestations of pregnancy rhinitis. One of the most affected processes is mucociliary clearance, which plays a key role in maintaining airway patency and protecting against pathogens. Studies suggest that the combination of mucosal edema, increased glandular secretion, and altered composition of nasal mucus during pregnancy may impair the normal ciliary beating frequency, leading to reduced mucociliary transport velocity [28]. As a result, patients may experience a persistent sensation of nasal obstruction and fullness, even in the absence of overt inflammation.

Neurophysiologically, autonomic dysregulation has been implicated in the pathogenesis of pregnancy rhinitis. Pregnancy is associated with increased parasympathetic activity, which promotes vasodilation and glandular hypersecretion in the nasal mucosa. This autonomic shift may enhance the effects of hormonal vasodilators such as estrogen and relaxin. Moreover, neuropeptides such as substance P and calcitonin gene-related peptide (CGRP)—known mediators of neurogenic inflammation—could play a role in amplifying vascular permeability and local reflex responses, although evidence specific to pregnancy rhinitis remains limited [29].

These neurogenic pathways may explain the enhanced vasodilation and mucosal reactivity seen in some pregnant women, despite the absence of overt inflammation or allergen exposure. The modulation of sensory nerve endings by estrogen and progesterone may also contribute to altered nociception and increased glandular activity, reinforcing the multifactorial nature of pregnancy rhinitis [28,29].

Changes in nasal airflow resistance are a well-documented functional consequence of mucosal congestion during pregnancy. A longitudinal cohort study demonstrated a significant rise in nasal airway resistance measured by anterior rhinomanometry from the first through the third trimester, which returned to baseline postpartum, confirming impaired nasal patency during gestation [30]. Moreover, a comparative study conducted in the third trimester showed that nearly 100% of pregnant women reported subjective nasal obstruction, with rhinomanometric data corroborating a marked increase in airflow resistance compared to non-pregnant controls (*p* < 0.001) [31].

It is also important to recognize that nasal obstruction during pregnancy may not be constant, but rather exhibit diurnal variation, with worsening at night or in the supine position. This pattern can be explained by the effects of gravitational redistribution of blood volume and enhanced venous pooling in the head and neck region during recumbency [32]. Furthermore, the lack of response to antihistamines in most cases of PR reinforces the notion that the underlying mechanism is not primarily allergic, but functional and hormonally mediated [33].

**Figure 2:**
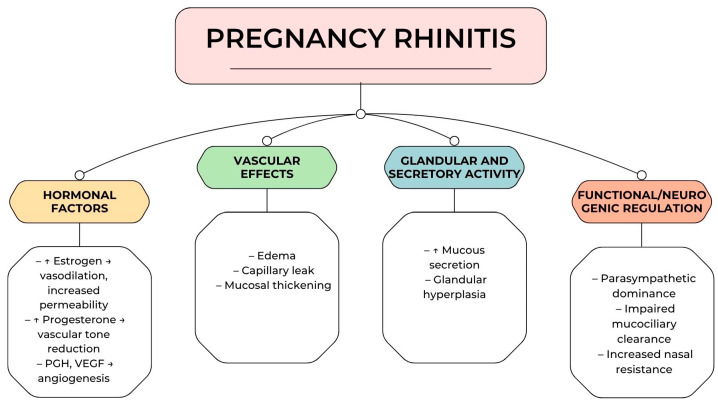
Multifactorial pathogenesis of pregnancy rhinitis.

The multifactorial nature of pregnancy rhinitis is summarized in Figure 2, which illustrates the main pathophysiological mechanisms contributing to the development of nasal congestion during pregnancy. Hormonal, vascular, glandular, and neurophysiological components interact to produce the clinical manifestations of the condition. Understanding this integrated model supports a more accurate diagnostic approach and guides tailored therapeutic strategies.

In summary, pregnancy rhinitis involves not only structural changes of the mucosa but also dynamic alterations in autonomic regulation, mucociliary function, and nasal airflow physiology. These functional impairments are essential to understanding the full clinical picture and help differentiate PR from allergic or infectious rhinitis, which are typically characterized by inflammatory or immunological features.

## 4. Clinical Features and Diagnostic Criteria

Pregnancy rhinitis typically manifests as persistent nasal congestion that arises during gestation, most frequently in the second or third trimester, and resolves spontaneously within two weeks postpartum. The condition is characterized by nasal obstruction without accompanying signs of infection (such as fever or purulent discharge) or evidence of allergic disease (e.g., sneezing, pruritus, conjunctival involvement) [1].

### 4.1. Clinical Manifestations

The hallmark symptom is bilateral nasal blockage, often more pronounced at night and in the supine position. Many patients report mouth breathing, nocturnal snoring, or even sleep disturbances due to impaired nasal airflow. Rhinorrhea may be present, but is typically mucous rather than serous or purulent. Other symptoms, such as postnasal drip or pressure in the nasal cavity, may occur but are generally mild. Importantly, systemic symptoms are absent, and the nasal mucosa appears non-inflamed or mildly edematous on anterior rhinoscopy [11].

The onset is often gradual, correlating with rising serum levels of estrogen and progesterone, and symptoms may worsen progressively as gestation advances. Patients usually do not report prior episodes of chronic rhinitis, and the resolution of symptoms shortly after delivery is a key diagnostic clue [34].

### 4.2. Clinical Criteria and Definitions

The diagnosis of pregnancy rhinitis remains clinical and is often established by exclusion, as no specific biomarker or imaging modality currently exists to confirm the condition. Several definitions have been proposed in the literature to standardize the identification of this entity, primarily in observational studies and expert consensus documents. Although variability persists, most authors agree on a core set of diagnostic elements that help differentiate pregnancy rhinitis from other forms of nasal obstruction [6,7].

Pregnancy rhinitis is typically defined as nasal congestion that

− Begins during pregnancy;
− Lasts for a minimum duration of six consecutive weeks;
− Is not associated with signs of respiratory infection (such as fever or purulent discharge);
− Occurs in the absence of allergic triggers;
− Resolves completely within two weeks postpartum [35].

These criteria aim to distinguish de novo rhinitis of pregnancy from exacerbations of pre-existing allergic, vasomotor, or structural nasal conditions. The timing of onset is particularly important, as symptoms that begin before conception or persist beyond the puerperium are less likely to represent true pregnancy rhinitis. Furthermore, the requirement of symptom resolution after delivery reinforces the presumed hormonal and physiological basis of the disorder [25].

Although not specifically addressed by ARIA (Allergic Rhinitis and its Impact on Asthma) guidelines, pregnancy rhinitis is often regarded as a distinct subtype of non-allergic, hormonally induced rhinitis, and its differentiation from allergic forms is important for clinical management [9].

Some authors have proposed classifying pregnancy rhinitis based on gestational age at onset (early vs. late) or severity of nasal obstruction, but these classifications are not universally adopted [1]. In clinical practice, a structured symptom history—combined with the exclusion of infection, allergy, and medication overuse—is usually sufficient to establish the diagnosis. Instruments such as the Nasal Obstruction Symptom Evaluation (NOSE) score or the Visual Analogue Scale (VAS) may be used to quantify symptom burden, although they are not specific to pregnancy-related rhinitis [36].

A clear understanding of the temporal profile and exclusion-based nature of this diagnosis is essential to avoid misclassification and to ensure appropriate counseling and management of affected patients.

### 4.3. Objective Evaluation Tools

Although the diagnosis of pregnancy rhinitis remains fundamentally clinical, a number of objective tools may support the assessment of symptom severity and the monitoring of disease progression. These instruments are particularly useful in research contexts or when evaluating the impact of therapeutic interventions.

One of the most commonly employed tools is the Nasal Obstruction Symptom Evaluation (NOSE) score, a patient-reported outcome measure composed of five items that address the functional consequences of nasal blockage. Each item is rated on a Likert scale, and the cumulative score provides a quantifiable index of symptom severity [36]. While not specific to pregnancy, this tool has been validated in various populations with nasal obstruction and is easy to apply in both outpatient and research settings.

The Visual Analogue Scale (VAS) is another widely used instrument, consisting of a 10-cm line on which patients mark their perceived level of nasal obstruction, ranging from no symptoms to maximal discomfort. The simplicity and reproducibility of this method make it suitable for longitudinal assessment, although it does not capture qualitative aspects of nasal airflow impairment [37].

While these tools can enhance the characterization of pregnancy rhinitis, they are not diagnostic in isolation. Their value lies in complementing the clinical evaluation and providing standardized measures to assess symptom burden or therapeutic efficacy, particularly in observational cohorts.

### 4.4. Differential Diagnosis

Given the nonspecific nature of nasal congestion and the high prevalence of other rhinitis subtypes, pregnancy rhinitis is frequently misdiagnosed or confused with more common nasal conditions [1]. A careful differential diagnosis is therefore essential to ensure that the obstruction is indeed pregnancy-induced and not the result of an unrelated or coexisting pathology.

Allergic rhinitis remains the most common alternative diagnosis. It is usually characterized by episodic symptoms triggered by exposure to allergens such as pollen, dust mites, or animal dander. Clinical features such as sneezing, nasal itching, watery rhinorrhea, and conjunctival irritation are typical. A personal or family history of atopy, seasonal variation in symptoms, and a positive response to antihistamines support the diagnosis. However, distinguishing allergic rhinitis from pregnancy rhinitis can be challenging in cases where both coexist, as hormonal changes may exacerbate allergic symptoms [38].

Non-allergic vasomotor rhinitis should also be considered, especially in patients with intermittent nasal obstruction triggered by environmental factors such as changes in temperature, humidity, or exposure to strong odors. This form of rhinitis is typically idiopathic, lacks inflammatory or allergic markers, and can present similarly to pregnancy rhinitis [39].

Rhinitis medicamentosa, resulting from prolonged or excessive use of topical nasal decongestants such as oxymetazoline or xylometazoline, must be excluded. Patients may report an initial period of symptom relief followed by rebound congestion and increased dependence on nasal sprays. This condition often requires active withdrawal of the causative agent and supportive treatment [40].

Infectious rhinitis or acute rhinosinusitis should be suspected when symptoms include purulent nasal discharge, facial pressure or pain, fever, and systemic malaise [41]. These features are not present in pregnancy rhinitis, and their presence should prompt further evaluation and, if necessary, antibiotic therapy.

Finally, structural abnormalities such as deviated nasal septum, turbinate hypertrophy, or nasal polyps may contribute to or mimic rhinitis symptoms. These are typically chronic in nature, often unilateral or asymmetric, and not limited to the gestational period. Nasal endoscopy may be indicated when structural causes are suspected, particularly in patients with persistent symptoms postpartum [42].

A comparative overview of key clinical features is presented in Table 1, highlighting the elements that help differentiate pregnancy rhinitis from other common causes of nasal obstruction, such as allergic rhinitis, rhinitis medicamentosa, and infectious rhinitis or acute rhinosinusitis.

In summary, differentiating pregnancy rhinitis from other forms of rhinitis relies on the temporal profile of symptoms, the absence of specific triggers or systemic signs, and the spontaneous resolution after delivery. A structured clinical assessment is essential for accurate diagnosis and appropriate management.

## 5. Impact on Maternal and Fetal Outcomes

Although pregnancy rhinitis is often perceived as a benign and self-limiting condition, its impact on maternal comfort, sleep quality, and possibly fetal well-being should not be underestimated. Persistent nasal obstruction can significantly affect the quality of life of pregnant women by impairing sleep, reducing daily functioning, and exacerbating fatigue and irritability during a period already associated with significant physiological stress [43].

One of the most commonly reported consequences of pregnancy rhinitis is disturbed sleep due to nasal congestion, particularly in the supine position. Studies have shown that women with moderate to severe nasal obstruction during pregnancy are significantly more likely to develop symptoms of sleep-disordered breathing—including snoring and sleep fragmentation—which can lead to nocturnal oxygen desaturation. In a systematic review, gestational rhinitis was explicitly linked to reduced sleep quality, increased snoring, and obstructive sleep apnea (OSA) [13,44]. Longitudinal data further indicate that the incidence of habitual snoring almost doubles, rising from 7 to 11% in the first trimester to 16–25% in the third trimester of pregnancy [45]. These findings suggest that pregnancy rhinitis may contribute to—or even unmask—gestational OSA, a condition now recognized as a potential risk factor for maternal hypertension, preeclampsia, and gestational diabetes.

From a fetal perspective, chronic maternal hypoxia and sleep disruption have been associated in some studies with adverse outcomes such as intrauterine growth restriction, low birth weight, or lower Apgar scores [46]. However, the evidence remains limited and largely indirect, as most studies have not isolated pregnancy rhinitis from other forms of sleep-disordered breathing or systemic maternal conditions. Nevertheless, given the potential for impaired oxygenation and the established physiological connections between maternal respiratory function and placental perfusion, attention to prolonged and symptomatic nasal obstruction is warranted [1,45].

In addition to respiratory effects, chronic nasal congestion in pregnant women may contribute to behavioral and emotional stress, particularly when sleep quality is affected. Multiparous women report significantly worse sleep quality, characterized by frequent nocturnal awakenings, fragmentation, and respiratory symptoms like snoring and nasal obstruction during the third trimester [47,48]. Poor sleep in late pregnancy has been correlated with a higher incidence of perinatal mood disturbances, including irritability, anxiety, and depressive symptoms [47,49]. These effects are likely compounded in women with multiple caregiving responsibilities, where disrupted sleep may also negatively impact daytime functioning and emotional well-being. Physiological data support that nasal congestion plays a significant role in sleep disturbance during pregnancy, especially in later gestation.

It is also important to consider the overuse of topical nasal decongestants [50], which some women may resort to in an attempt to alleviate persistent nasal symptoms. Prolonged use of vasoconstrictive agents such as oxymetazoline carries the risk of developing rhinitis medicamentosa and may have theoretical systemic effects on uteroplacental blood flow. While occasional short-term use may be acceptable in the second or third trimester under medical supervision, chronic use should be avoided and alternatives considered [51,52].

Despite these potential consequences, pregnancy rhinitis is rarely a direct cause of serious obstetric complications. Nonetheless, its indirect effects on maternal well-being, sleep physiology, and potential fetal oxygenation merit greater clinical attention. Early recognition and symptomatic management may contribute to improved quality of life and support optimal maternal–fetal health.

## 6. Management Strategies

The management of pregnancy rhinitis poses a clinical challenge due to its unclear pathophysiology, the self-limiting nature of the condition, and concerns regarding the safety of pharmacological agents during gestation. As no specific treatment targets the underlying hormonal and vascular mechanisms, therapeutic strategies focus primarily on symptom relief and quality-of-life improvement, while minimizing risks to both mother and fetus [9].

### 6.1. Non-Pharmacological Measures

First-line management typically includes non-pharmacological approaches that aim to reduce mucosal edema, enhance nasal airflow, and improve sleep. These interventions are considered safe throughout pregnancy and may be sufficient in mild to moderate cases. Saline nasal irrigation is widely recommended as a mechanical method to clear mucus, reduce nasal dryness, and restore mucociliary function. Both isotonic and hypertonic saline solutions have been used, with some studies suggesting that hypertonic formulations may offer greater short-term decongestion [53]. Some commercially available irrigation solutions also contain additives such as xylitol or essential oils; however, their safety profiles during pregnancy are not well established, and plain saline (isotonic or hypertonic) remains the recommended option in this population [53].

Use of external nasal dilator strips can be beneficial, particularly at night, by mechanically widening the nasal valve area and reducing inspiratory resistance. They are non-invasive, drug free, and have demonstrated efficacy in improving sleep quality in pregnant women with nasal obstruction [54].

Humidification of ambient air, especially during colder seasons or in air-conditioned environments, may help reduce mucosal irritation and congestion. Positional therapy, such as sleeping in a semi-upright position, may further improve nocturnal airflow [55]. Lifestyle modifications, including weight management in cases of high BMI and smoking cessation, are also important. Smoking not only exacerbates mucosal inflammation but is independently associated with poor maternal–fetal outcomes and should be strongly discouraged [56].

Patient education is essential, as many women may be unaware that their symptoms represent a distinct clinical entity. Reassurance regarding the benign nature of the condition and its resolution postpartum can improve compliance and reduce anxiety.

### 6.2. Pharmacological Approaches

In moderate to severe cases where non-pharmacological measures are insufficient, pharmacologic therapy may be considered, with careful evaluation of the risk–benefit ratio. Medication use in pregnancy is limited by safety concerns, especially during the first trimester.

Topical nasal corticosteroids, such as budesonide and fluticasone, are considered relatively safe and are categorized as class B drugs by the FDA. Budesonide, in particular, has the most robust safety data in pregnancy and may be used to reduce mucosal inflammation and edema [57]. However, evidence for their efficacy in pregnancy rhinitis specifically is limited.

Second-generation oral antihistamines, including loratadine and cetirizine, may be used in cases with overlapping allergic symptoms or unclear etiology. These agents have favorable safety profiles and do not cause significant sedation. Their role in pure pregnancy rhinitis, however, remains marginal, as histamine is not a central mediator in this condition [58].

Topical nasal decongestants such as oxymetazoline may offer rapid relief but should be used with caution. A single short-term dose does not significantly alter maternal–fetal blood flow [59], and limited studies suggest that usage in the second or third trimester may be safe if confined to a brief duration (≤10 days) [60]. However, prolonged use beyond three to seven days has been clearly associated with the development of rhinitis medicamentosa—a rebound nasal congestion syndrome. Therefore, their use should be strictly limited to short-term relief, ideally before sleep, and always under medical supervision [51].

Intranasal anticholinergics (e.g., ipratropium bromide) and cromolyn sodium have limited data in pregnancy and are not routinely recommended unless benefits clearly outweigh potential risks [61].

The safety of pharmacological agents during pregnancy is often assessed using the FDA (U.S. Food and Drug Administration) classification system, which categorizes medications based on their potential risk to the fetus. Category B includes drugs for which animal studies have shown no fetal risk and there are no adequate human studies, or where animal studies showed adverse effects not confirmed in humans. Category C is assigned when animal studies have shown adverse effects and no adequate studies exist in humans; however, potential benefits may justify their use [62,63]. These categories have been included in Table 2 for clarity, while non-pharmacological measures are not subject to FDA classification.

An overview of the main treatment options available for pregnancy rhinitis is provided in Table 2, including both non-pharmacological and pharmacological approaches. The table summarizes each intervention by category, evaluates its safety profile during pregnancy, and offers practical notes regarding its clinical use. This structured comparison may serve as a guide for individualized therapeutic decisions based on symptom severity, gestational age, and maternal preference.

In all cases, therapeutic decisions should be individualized, taking into account symptom severity, gestational age, patient preferences, and comorbid conditions. Coordination between obstetricians and ENT specialists can be beneficial in complex or refractory cases.

## 7. Current Gaps and Future Directions

Despite increasing recognition of pregnancy rhinitis as a distinct clinical entity, several knowledge gaps persist, limiting both the understanding of its pathophysiology and the development of evidence-based management strategies.

One major limitation is the lack of standardized diagnostic criteria. Existing definitions rely on the exclusion of other rhinitis types and postpartum resolution, but there is no universally accepted clinical or laboratory marker to confirm the diagnosis. This ambiguity complicates epidemiological comparisons across studies and hinders the development of targeted treatment trials.

The precise pathophysiological mechanisms remain incompletely elucidated. Although hormonal, vascular, and neurogenic factors are strongly implicated, the specific contributions of each remain speculative. There is a lack of histological or biomolecular studies exploring receptor expression, mucosal remodeling, or inflammatory profiles in affected versus unaffected pregnant women. Similarly, genetic or epigenetic susceptibility factors have not been systematically investigated.

Another critical gap involves therapeutic research. Most current recommendations are extrapolated from studies on allergic or vasomotor rhinitis, with very few randomized controlled trials specifically addressing treatment efficacy or safety in pregnancy rhinitis. The role of nasal corticosteroids, antihistamines, or non-pharmacological therapies has not been rigorously evaluated in this population. Furthermore, concerns over fetal safety continue to limit the use of pharmacologic interventions, even in cases where symptom burden is substantial.

The impact of pregnancy rhinitis on maternal health outcomes—such as sleep quality, mood disturbances, and fatigue—has been documented in small studies but remains under-investigated. Even more uncertain is the extent to which this condition contributes to fetal outcomes through mechanisms related to maternal hypoxia or poor sleep. Prospective studies with objective sleep and respiratory monitoring could help clarify these associations.

Future research should prioritize the development of clear diagnostic criteria, including potential biomarkers or objective clinical scoring systems. Large-scale, prospective cohort studies are needed to assess the true prevalence, natural history, and long-term implications of pregnancy rhinitis. Controlled trials should also evaluate both pharmacologic and non-pharmacologic interventions, with a focus on maternal quality of life and fetal safety.

Interdisciplinary collaboration between obstetrics, otorhinolaryngology, and sleep medicine may be essential to advance the understanding and management of this condition. With a growing emphasis on personalized prenatal care, pregnancy rhinitis deserves greater clinical attention and dedicated research efforts.

## 8. Conclusions

Pregnancy rhinitis is a clinically relevant but frequently underdiagnosed condition that affects a significant proportion of pregnant women. Although symptoms are typically mild to moderate and self-limited, their impact on sleep quality, daily functioning, and overall maternal well-being can be substantial. The pathogenesis involves a complex interplay of hormonal, vascular, glandular, and neurophysiological factors, yet the exact mechanisms remain incompletely understood.

Diagnosis is based on clinical criteria and requires exclusion of other causes of nasal obstruction. While non-pharmacological measures form the cornerstone of management, selected pharmacologic agents may be used safely in moderate to severe cases. Increased awareness among healthcare providers and clear communication with patients are essential to ensure appropriate recognition and supportive care.

There remains a need for standardized diagnostic definitions, high-quality clinical trials, and mechanistic studies to better understand and manage this condition. A multidisciplinary approach involving obstetricians, ENT specialists, and sleep medicine experts may help optimize outcomes for both mother and fetus. By addressing the current gaps in knowledge and promoting targeted research, pregnancy rhinitis can transition from a neglected discomfort to a recognized and manageable component of prenatal care.

## Figures and Tables

**Table 1:** Comparative features of pregnancy rhinitis, allergic rhinitis, rhinitis medicamentosa, and infectious rhinitis.

Feature	Pregnancy Rhinitis [35]	Allergic Rhinitis [38]	Rhinitis Medicamentosa [40]	Infectious Rhinitis [25]
Onset	2nd–3rd trimester	Seasonal or perennial	After prolonged nasal spray use	Sudden, often post-viral
Duration	≥6 weeks during pregnancy	Variable, often chronic	Progressive with drug dependence	7–10 days typically
Resolution	≤2 weeks postpartum	Persists unless treated	Persists unless medication stopped	Resolves spontaneously or with antibiotics
Nasal discharge	Mucous	Clear, watery	Often absent or rebound congestion	Purulent or mucopurulent
Sneezing and pruritus	Absent	Common	Absent	Variable
Systemic symptoms	Absent	Rare	Absent	Common (fever, fatigue)
Response to antihistamines	Minimal	Good	None	None
Prior rhinitis history	Usually absent	Often positive	Often negative	Often negative

**Table 2:** Overview of treatment modalities for pregnancy rhinitis.

Treatment Option	Category	Safety in Pregnancy	Notes	FDA Category
Saline nasal irrigation [53]	Non-pharmacologic	Safe in all trimesters	Improves mucociliary clearance	Not applicable
Nasal dilator strips [54]	Non-pharmacologic	Safe	Beneficial at night	Not applicable
Humidified air [55]	Non-pharmacologic	Safe	Reduces mucosal dryness	
Sleeping with elevated head [55]	Non-pharmacologic	Safe	Reduces nocturnal congestion	Not applicable
Budesonide nasal spray [57]	Pharmacologic	Safe (Category B)	First-line intranasal steroid	B
Fluticasone nasal spray [57]	Pharmacologic	Likely safe	Use after risk-benefit assessment	C
Cetirizine, loratadine [58]	Pharmacologic	Safe after 1st trimester	Use in allergic overlap only	B
Oxymetazoline spray (short-term) [56,57]	Pharmacologic	Use with caution	Limit to 3–5 days; avoid in 1st trimester	C
Ipratropium nasal spray [58]	Pharmacologic	Limited data	Not routinely recommended	B/C
